# Dietary β-hydroxybutyric acid improves the growth performance of young ruminants based on rumen microbiota and volatile fatty acid biosynthesis

**DOI:** 10.3389/fmicb.2023.1296116

**Published:** 2024-01-08

**Authors:** Jianmin Chai, Zeyue Liu, Jun Wu, Yuan Kang, Mahmoud M. Abdelsattar, Wei Zhao, Shiqin Wang, Shuli Yang, Feilong Deng, Ying Li, Yimin Zhuang, Naifeng Zhang

**Affiliations:** ^1^Guangdong Provincial Key Laboratory of Animal Molecular Design and Precise Breeding, College of Life Science and Engineering, Foshan University, Foshan, China; ^2^Key Laboratory of Feed Biotechnology of the Ministry of Agriculture and Rural Affairs, Institute of Feed Research of Chinese Academy of Agricultural Sciences, Beijing, China; ^3^Division of Agriculture, Department of Animal Science, University of Arkansas, Fayetteville, NC, United States; ^4^Foshan Hospital of Traditional Chinese Medicine, Foshan, China; ^5^Department of Animal and Poultry Production, Faculty of Agriculture, South Valley University, Qena, Egypt; ^6^Anhui Province Key Laboratory of Animal Nutritional Regulation and Health, College of Animal Science, Anhui Science and Technology University, Chuzhou, China; ^7^State Key Laboratory of Animal Nutrition, College of Animal Science and Technology, China Agricultural University, Beijing, China

**Keywords:** β-hydroxybutyric acid, rumen microbiota, goats, growth, volatile fatty acids

## Abstract

**Introduction:**

The ketone body β-hydroxybutyric acid (BHB) plays critical roles in cellular proliferation and metabolic fuel utilization; however, its effects on the rumen microbiota remain unknown.

**Methods:**

Here, three doses of BHB (low, medium, and high) were supplemented to early-weaned goat kids.

**Results:**

Compared with controls, the beneficial effects of BHB on growth and rumen development were observed in goats at 90 days of age (d). The low dose of dietary BHB increased the concentration of rumen acetate, propionate, and butyrate on d90. The sequencing results of the rumen microbiota revealed marked shifts in rumen microbial community structure after early-weaned goat kids consumed BHB for 2 months. The signature bacterial ASVs for each treatment were identified and were the main drivers contributing to microbial interactions in the rumen. The bacteria associated with rumen weight were also correlated with body weight. Some classified bacterial signatures, including *Prevotella, Olsenella umbonate*, and *Roseburia faecis*, were related to rumen volatile fatty acids and host development.

**Conclusion:**

Overall, dietary BHB altered rumen microbiota and environments in young goats, which contributed to rumen development and growth.

## Implications

Significant improvements in rumen volatile fatty acid biosynthesis and development were observed by BHB supplementation, resulting in increased animal growth. Using sequencing technology, this study confirmed that BHB supplementation altered rumen microbiota in a distinct manner, and BHB dose could act in different mechanisms to influence the microbiota. Dietary BHB could increase some specific bacteria and drive their interactions with other core microbiota. These abundant microbiotas and their metabolites improved the utilization efficiency of ketone bodies to improve rumen and host development. This study provides a landmark reference for the future use of BHB to improve animal growth performance.

## Introduction

High-quality proteins from ruminants are one of the most essential sources of human consumption. In ruminants, the rumen, as the critical organ, converts the dietary plant materials into high-quality proteins through microbiome fermentation (Mizrahi et al., 2021). The fermented microbial products [i.e., volatile fatty acids (VFAs)] not only provide energy for the host but also are associated with the development of the rumen epithelium (Lin et al., 2019; Malmuthuge et al., 2019; Chai et al., 2021). A well-developed rumen in young ruminants associated with early colonization and development of the rumen microbial community is important and has long-term impacts on growth performance, nutrient utilization, and lifelong health (Wang et al., 2019; Zhuang et al., 2020). Therefore, understanding interactions between the rumen microbiota and ruminant host in early life is essential for the sustainable development of the livestock industry (Matthews et al., 2019; Morais and Mizrahi, 2019; Zhuang et al., 2023b).

The ketogenic capacity of the ruminal epithelium, one of the most important parameters, that represents the maturation of rumen functions, is important and less studied (Lv et al., 2019; Lin et al., 2020; Zhuang et al., 2020). Rumen VFAs could diffuse into rumen epithelial cells and are metabolized into ketones which provide energy and facilitate signals for the host gene transcription and the regulation of metabolism. The process in which acetyl-CoA from butyrate is converted to the ketone body (e.g., β-hydroxybutyric acid (BHB) and acetoacetic acid) is called ketogenesis (Abdelsattar et al., 2022a). BHB, the major ketones from the metabolism of VFA in rumen epithelium, might be the biomarkers for the maturation of rumen functions and microbiota as well as VFA utilization ability (Deelen et al., 2016). Beyond its role as an energy carrier, Cheng et al. found that BHB increased signaling to bolster self-renewing capabilities (i.e., proliferation and function) in mice intestinal stem cells (Cheng et al., 2019). Another study observed that BHB contributes to intestinal cell differentiation in mice (Wang et al., 2017). Our previous research also confirmed the positive effects of exogenous BHB on rumen and body weight in young goats (Abdelsattar et al., 2022a).

Dietary BHB may, in part, affect the gut microbiota and then benefit the host. A recent study found that supplementation of oral ketone esters (mainly composed of BHBA) significantly altered the gut microbiota in mice and humans, and BHB inhibited the growth of *ex vivo* fecal microbiota in a dose-dependent manner (Ang et al., 2020). Another *in vitro* study concluded that human colonic microbiotas could efficiently utilize BHB to increase butyrate production (Sasaki et al., 2020). However, to the best of our knowledge, there is no research that investigates the impacts of dietary BHB on the rumen microbiota in ruminants.

We hypothesized that BHB supplementation could partially act *via* the rumen microbiota and improve the rumen development and host growth. In this study, rumen microbiota from goat kids consuming different doses of BHB was sequenced, and the changes in rumen bacteria caused by BHB were identified. Our results provide a view of the mechanistic links between BHB, microbiota, and host physiology.

## Materials and methods

In 2020, this study was carried out at an experimental base goat farm in Jiangsu, China. All experimental procedures were approved by the Animal Ethics Committee of the Chinese Academy of Agricultural Sciences (protocol number: AEC-CAAS-20200605; approval date: 3 June 2020).

### Animals, treatments, and sampling collection

Sixty-four goat kids (5.14 ± 1.08 kg of body weight) were separated from their dams at 30 days of age and selected for this study. The kids were assigned to four treatments in a completely randomized design (eight replicates per treatment). The solid diet (starter) supplemented with 0 (control, C), 3 (low dose, L), 6 (medium dose, M), or 9 (high dose, H) g/d per animal; β-hydroxybutyric acid (BHB) was provided to the experimental goat kids from 30 to 90 days (d) of age. At d 90, blood samples were collected, and six goat kids from each treatment were slaughtered for the rumen sample collection, resulting in four groups, namely, C90, L90, M90, and H90.

The BHB was composed of BHB Na with a purity of 99% (Wuhan Huajiu Pharmaceutical Technology Co., Ltd., China). The animal trial lasted for 2 months. All the goat kids were housed separately in 32 covered pens (2 kids for each pen as one replicate) with a slatted floor (2 m^2^), feeders, and water buckets. The body weight was recorded at d30 and d90. The early-weaned kids were provided with a designed milk replacer from d30 to d60 (the feeding amount of milk replacer was based on 2% of body weight). During the trial, all animals had free access to a solid commercial starter. The chemical compositions of the diet are displayed in Supplementary Table S1. At the end of the trial, blood samples were collected into 10 ml vacutainer tubes without anticoagulants using the jugular venipuncture method. Then, they were centrifugated at 3,000 rpm for 10 min, and the supernatant serum was transferred to 1.5 ml Eppendorf tubes for BHB determination. Then, goat kids were slaughtered at the abattoir of the goat farm based on our previous methods (Chai et al., 2015). Approximately 10 ml of rumen content was sampled and stored at −80°C for the microbial sequencing. In total, 10 ml of rumen fluid was filtered via four layers of gauze and immediately frozen at −20°C for the analysis of rumen fermentation parameters and BHB.

### Serum and rumen BHB determination

The biochemical analyses of BHB in goat serum samples were performed at the laboratory of Beijing Jinhai Keyu Biotechnology Development Co., Ltd., Beijing, China. In brief, the blood BHB concentrations were determined using an ELISA commercial kit (Beijing Jinhai Keyu Biotechnology Development Co., Ltd.), according to the manufacturer's instructions. During the trial, no cross-reactions were observed with other soluble structural analogs. All coefficients of variation for intra-assay and inter-assay were smaller than 10%. In addition, the assay sensitivity was 0.1 mmol/L for BHB.

The BHB concentration in the rumen was measured based on a previous method (Abdelsattar et al., 2022a). In brief, we used 1 ml of ice-cold methanol [80% v/v, 0.1% formic acid] to extract samples, and the subsequent step was homogenization. Then, after centrifugation, supernatants were dried in a vacuum and reconstituted in 100 ml of methanol (3% v/v). Quantitative analysis was performed on the LC-MS system controlled by Xcalibur 2.2 software (all from Thermo Fisher Scientific, Waltham, MA). Targeted analytes were measured and normalized by a 13C stable isotope-labeled internal standard (Cambridge Isotope Laboratories, Inc., Tewksbury, MA). Finally, the results were calculated based on a standard curve and its concentrations ranged from 0.1 mM to 50 mM.

### Measurement of rumen VFAs

Regarding the measurements of VFA concentrations in the rumen, samples with metaphosphoric acid were thawed at room temperature and then centrifuged (12,000 × *g* for 15 min at 4°C). The supernatant was loaded into gas chromatography (GC) (Jiao et al., 2014). The GC used methyl valerate as the internal standard in an Agilent 6890 series GC equipped with a capillary column (HP-FFAP19095F-123, 30 m, 0.53 mm diameter, and 1 mm thickness, Agilent Technologies, Santa Clara, CA, USA).

### Rumen microbial sequencing

The total genomic DNA of rumen content samples was extracted using a DNeasy PowerSoil Kit (Qiagen, Valencia, CA, USA), based on the manufacturer's recommendation. The DNA concentration and quality were determined using a Thermo NanoDrop 2000 UV microphotometer and 1% agarose gel electrophoresis. The genomic DNA was diluted to the optimal concentration and used as a template for PCR. The V3–V4 region of bacterial DNA was amplified using adaptor-linked universal primers (341F: CCTACGGGRSGCAGCAG; 806R GGACTACVVGGGTATCTAATC). Following the manufacturer's instructions for the KAPA HiFi Hotstart ReadyMix PCR Kit, PCR was performed to amplify DNA. Next, the AxyPrep DNA Gel Recovery Kit (AXYGEN Inc., Union City, CA, USA) was used to cut the gel and obtain the PCR products. To prevent reagent contamination, negative controls were included during the process of DNA extraction and PCR amplification, and no PCR products of the negative controls were observed from agarose gel. Then, all PCR products were mixed to make the sequencing library, and their quality was checked using both a Thermo NanoDrop 2000 UV spectrophotometer and 2% agarose gel electrophoresis. A Qubit 2.0 Fluorometer (Thermo Fisher Scientific, Waltham, MA, USA) was used to quantify the library. An Illumina Miseq PE250 platform (Realbio Technology Genomics Institute, Shanghai, China) was used to sequence the prepared amplicon library.

### Bioinformatics and statistics

Raw sequencing data were processed using the QIIME2 platform (2021.4 release) (Bolyen et al., 2019). The Deblur integrated with QIIME2 was employed to process the demultiplexed sequences with the default parameter setting (Amir et al., 2017). The detailed steps contained paired read merging, barcode and length trimming, quality filtering, denoising (Deblur), bacterial classification (Greengenes reference database; 13–8 version; 99% similarity), and sequence clustering. Chimeric sequences and singletons were removed for downstream analysis based on the official pipeline, and amplicon sequence variants (ASVs) were clustered based on 100% identity. Then, the number of reads of all samples was rarefied based on the minimum depth to reduce the errors of sequencing depth on alpha (Shannon Index and Observed ASVs) and beta (Bray–Curtis and Jaccard) diversity measures. Analysis of similarity (ANOSIM) was used to test the differences in beta diversity in QIIME2. The sequencing data of the current study are available in the NCBI BioProject database with the BioProject ID PRJNA932733.

The body weight, serum and rumen BHB, and rumen fermentation parameters were displayed using boxplots made in R (v3.6.0) by the ‘ggplot2′ package. ANOVA tests were performed to test the significance after detecting homogeneity of variance. After checking the global test significance, a *post-hoc* analysis (Tukey's HSD test) was performed to estimate which groups of independent variables differ from the other groups.

Alpha diversity, including the Shannon Index and the number of observed ASVs, was analyzed for differences using the Wilcoxon rank-sum test in RStudio (RStudio Inc., Boston, MA). Based on the Bray–Curtis and Jaccard distances, beta diversity was visualized on the principal coordinate analysis (PCoA) plot. The stacked bar plot for microbial composition at the phylum and genus levels was drawn in R. Differentially represented bacterial ASVs between groups were identified using galaxy linear discriminant analysis (LDA) effect size (LEfSe) with default settings (e.g., LDA score > 2) (Segata et al., 2011).

Network analysis of bacterial interactions was performed. Using the R package “psych”, the Spearman correlations (r) among the core bacterial ASVs with an average abundance over 0.1% were calculated to reveal the correlation between rumen bacteria at the ASV level, with an absolute correlation coefficient over 0.6 and *p*-values smaller than 0.05. Then, Gephi software was used to visualize co-occurrence patterns of rumen bacteria (Girvan and Newman, 2002).

Regression-based Random Forest algorithm was used to select the rumen microbiota to predict the host phenotypes (i.e., body weight, rumen weight, and VFAs). The top 50 selected features were then analyzed by Pearson's correlation with those macro indicators, respectively.

## Results

### Dietary β-hydroxybutyric acid (BHB) improved growth and rumen environment in goat kids

**Figure 1:**
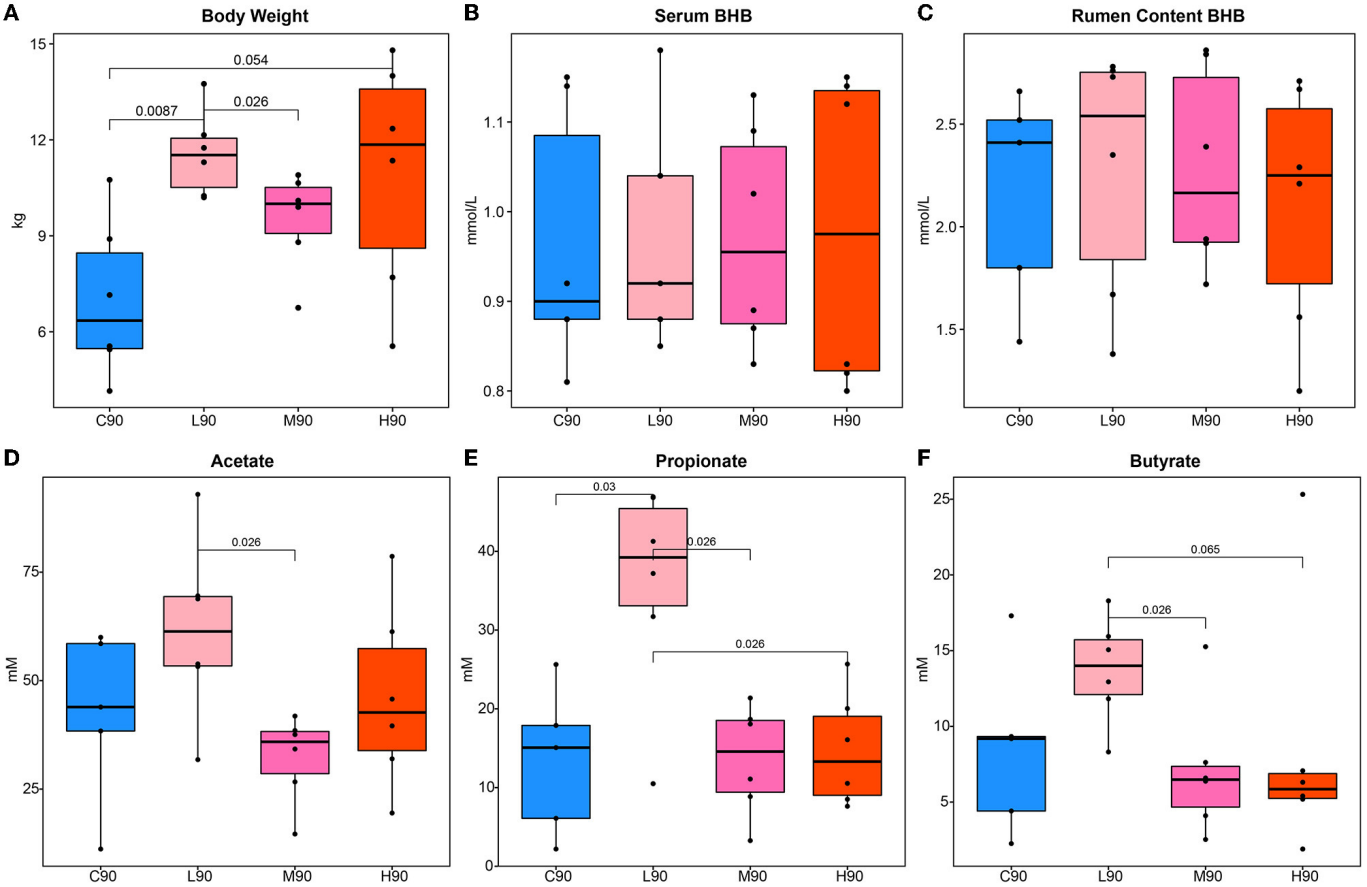
Dietary β-hydroxybutyric acid (BHB) improved growth and rumen environment in goat kids. Different levels of dietary BHB supplementation (low, medium, and high) increased body weight **(A)** in goat kids at 90 days of age but did not affect the concentration of BHB in serum **(B)** and rumen content **(C)** compared to the control group. The low dose of BHB supplementation increased rumen acetate **(D)**, propionate **(E)**, and butyrate **(F)**. An ANOVA test was used for significance calculation after detecting homogeneity of variance. After the global test was significant, a *post-hoc* analysis (Tukey's HSD test) was performed to determine which group of the independent variable differed from each other group. Significant *p*-values were labeled over the bars. C90 = controls at 90 days of age; L90: low dose (3 g/d per animal BHB) at 90 days of age; M90: medium dose (6 g/d per animal BHB) at 90 days of age; H90: high dose (9 g/d per animal BHB) at 90 days of age.

At d90, significant increases in the body weight of goat kids by dietary BHB supplementation were observed in the L90 and H90 groups compared with controls (Figure 1A). The value of M90 body weight was higher than C90, but no statistical difference was observed. Interestingly, the body weight in M90 was lower than in L90. Similarly, increases in the rumen weight in L90 than in C90 were found (Supplemenatry Figure S1). The M90 and H90 had higher rumen weight compared with controls, while they were not significant. Then, we also observed that the levels of circulating and rumen content BHB were not different (Figures 1B, C). Next, rumen fermentation parameters were measured to illustrate the effect of dietary BHB on the rumen environment (Figures 1D–F). The low dose of BHB supplementation increased rumen acetate, propionate, and butyrate. L90 had a higher concentration of acetate than M90, while the concentration of propionate in L90 was more significant than in the other groups. At the same time, butyrate in L90 was higher compared with M90 and H90. Notably, there were no differences in rumen fermentation parameters among C90, M90, and H90.

### Dietary β-hydroxybutyric acid altered the diversity and structure of the rumen microbiota in goat kids

**Figure 2:**
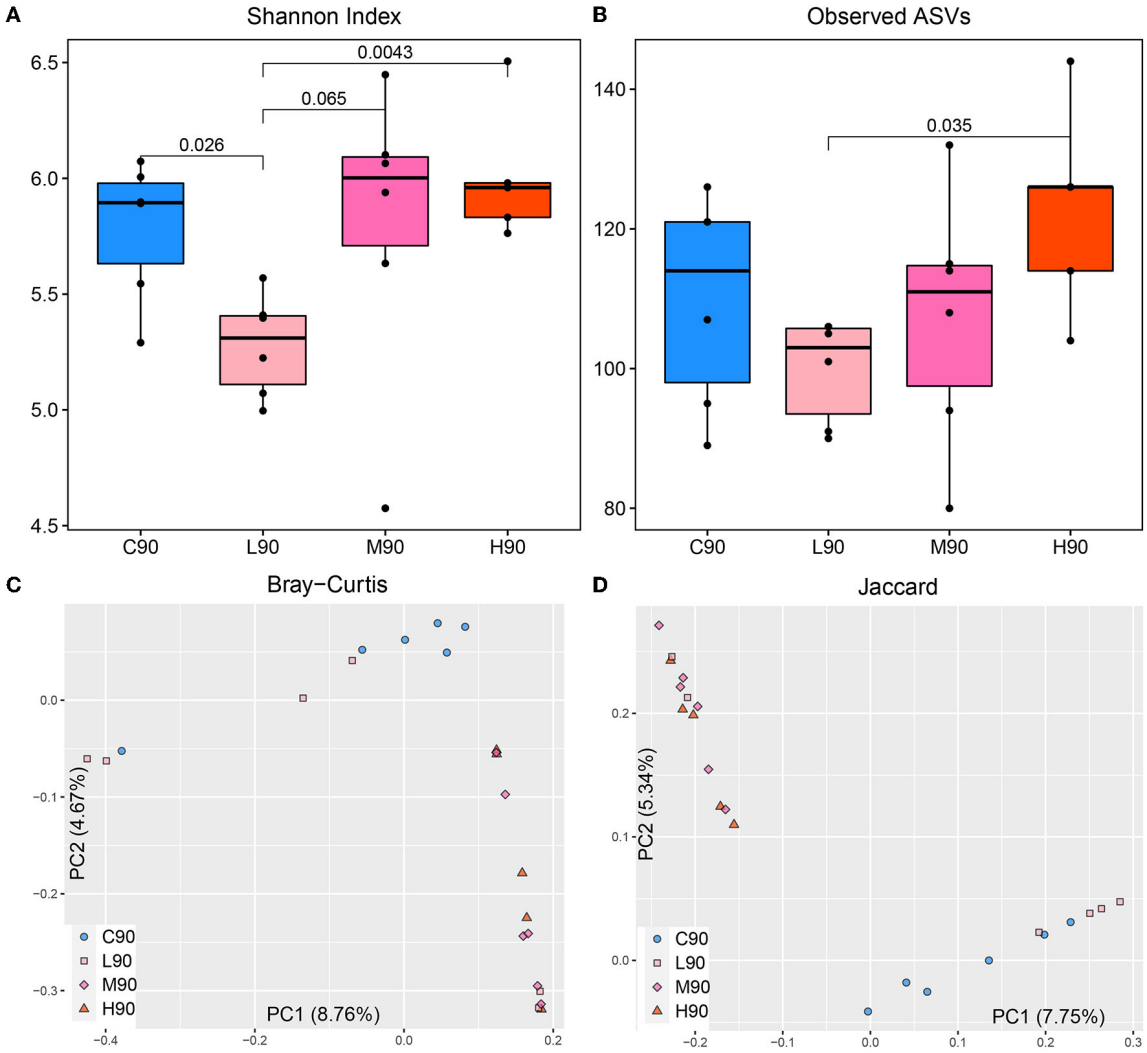
Dietary β-hydroxybutyric acid (BHB) altered alpha and beta diversity of the rumen microbiome in goat kids. **(A, B)**: The Shannon Index and the number of observed ASVs of rumen microbiota. The line inside the box denotes the median, and the boxes denote the interquartile (IQR) between the first and third quartiles (25th and 75th percentiles, respectively). The significance among sampling time points calculated using the Wilcoxon rank-sum test was labeled. **(C, D)**: Principal Coordination Analysis (PCoA) of Bray-Curtis and Jaccard distances for microbiota. Each point represents a unique sample. C90 = controls at 90 days of age; L90: low dose (3 g/d per animal BHB) at 90 days of age; M90: medium dose (6 g/d per animal BHB) at 90 days of age; H90: high dose (9 g/d per animal BHB) at 90 days of age.

A total of 101,022 high-quality reads at the single-nucleotide resolution were generated. After the rarefaction of sample reads to a minimum, 2,537 ASVs were included for downstream analysis of the rumen microbial community. Regarding alpha diversity, the Shannon index in L90 was significantly lower compared with C90, M90, and H90, which were not different (Figure 2A). The number of observed ASVs in L90 was lower than in H90 (Figure 2B). Regarding beta diversity measurements, significant clusters in community structure among four groups were detected (Figures 2C, D). Compared with C90, L90, M90, and H90 clustered separately based on Bray–Curtis (ANalysis Of SIMilarity (ANOSIM), R-value: C90–L90 = 0.17, C90–H90 = 1.00, and C90–H90 = 1.00, *p* < 0.05) and Jaccard distances (ANOSIM, R-value: C90-L90 = 0.19, C90-H90 = 1.00, and C90-H90 = 1.00, *p* < 0.05). Similarly, compared with L90, M90 and H90 showed distinct clusters based on both Bray–Curtis (ANOSIM, R-value: L90–M90 = 0.40, and L90–H90 = 0.37, *p* < 0.05) and Jaccard distances (ANOSIM, R-value: L90-M90 = 0.42, and L90–H90 = 0.42, *p* < 0.05). However, no different cluster between M90 and H90 was observed.

**Figure 3:**
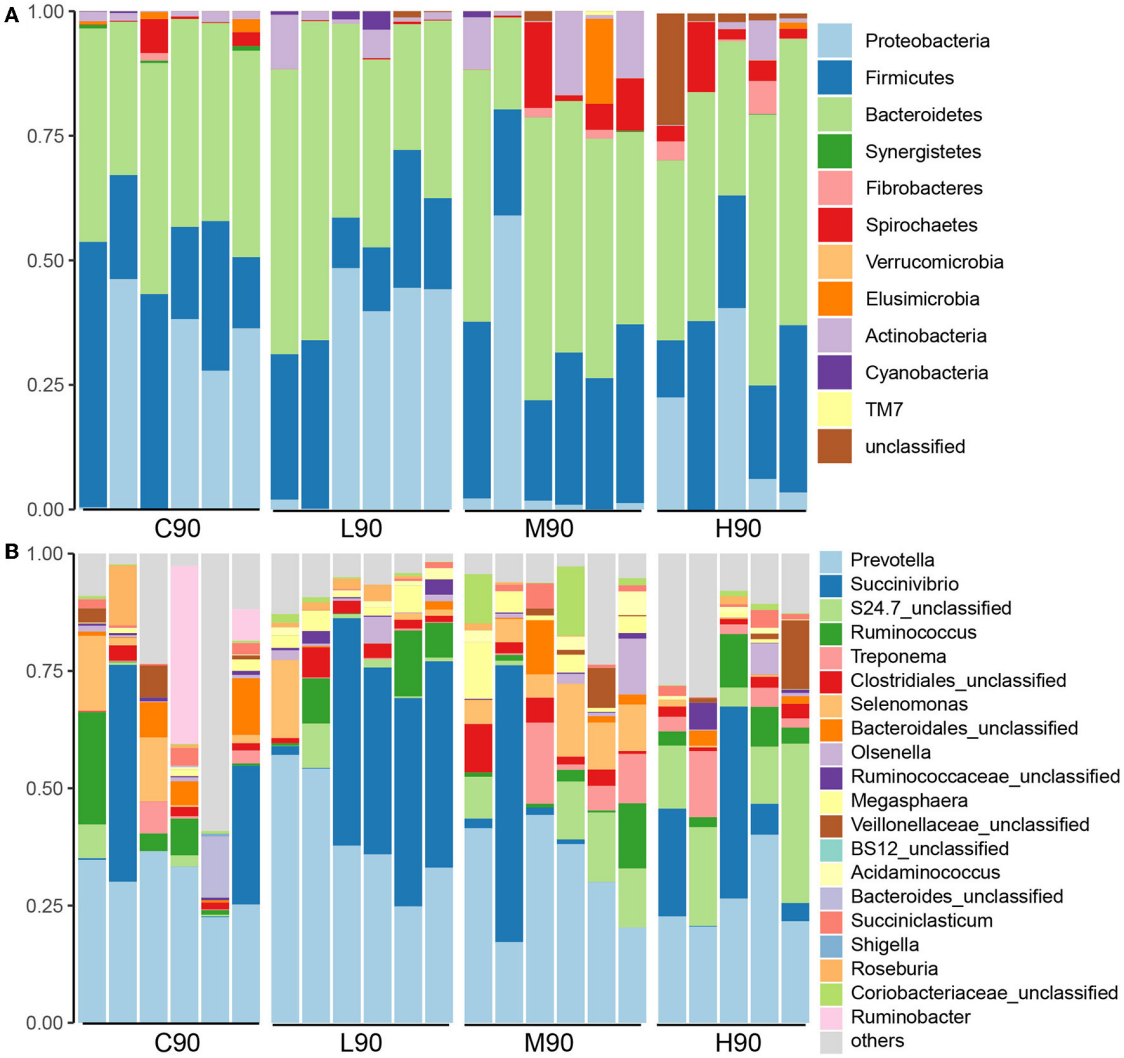
Microbial compositions were associated with dietary β-hydroxybutyric acid (BHB). Stacked bar charts showed that different levels of dietary BHB supplementation (low, medium, and high) altered the rumen microbial composition at the phylum level **(A)** and the genus level **(B)** in 90-day-old goat kids. Each column represents one sample. C90 = controls at 90 days of age; L90: low dose (3 g/d per animal BHB) at 90 days of age; M90: medium dose (6 g/d per animal BHB) at 90 days of age; H90: high dose (9 g/d per animal BHB) at 90 days of age.

At the phylum level, Bacteroidetes, Firmicutes, and Proteobacteria were dominant phyla across all samples accounting for 89% of the total sequences (Figure 3A). Although dietary BHB supplementation did not influence the relative abundance of Bacteroidetes, Firmicutes was lower in L90 (21.98%), M90 (28.28%), and H90 (24.90%) compared with C90 (30.02%). The abundance of Proteobacteria in M90 (10.88%) and H90 (14.90%) was lower than that in L90 (24.88%) and L90 (29.85%). At the genus level, *Prevotella, Succinivibrio, S24.7 unclassified*, and *Ruminococcus* were the dominant genera (Figure 3B). The abundance of *Prevotella* (C90: 30.42%, L90: 40.49%, M90: 31.92% and H90: 26.30%) was influenced by dietary BHB. A higher abundance of *Succinivibrio* in L90 (29.78%) was observed compared with C90 (12.73%), M90 (10.60%), and H90 (14.90%). Medium and high doses increased the relative abundance of *Treponema* and *Olsenella*. In addition, the relative abundance of the *Selenomonas* genus was increased in M90 (8.42%) but decreased in L90 (3.23%) and H90 (0.44%) compared with the control (5.54%).

### The signature rumen microbiota associated with dietary BHB supplementation

**Figure 4:**
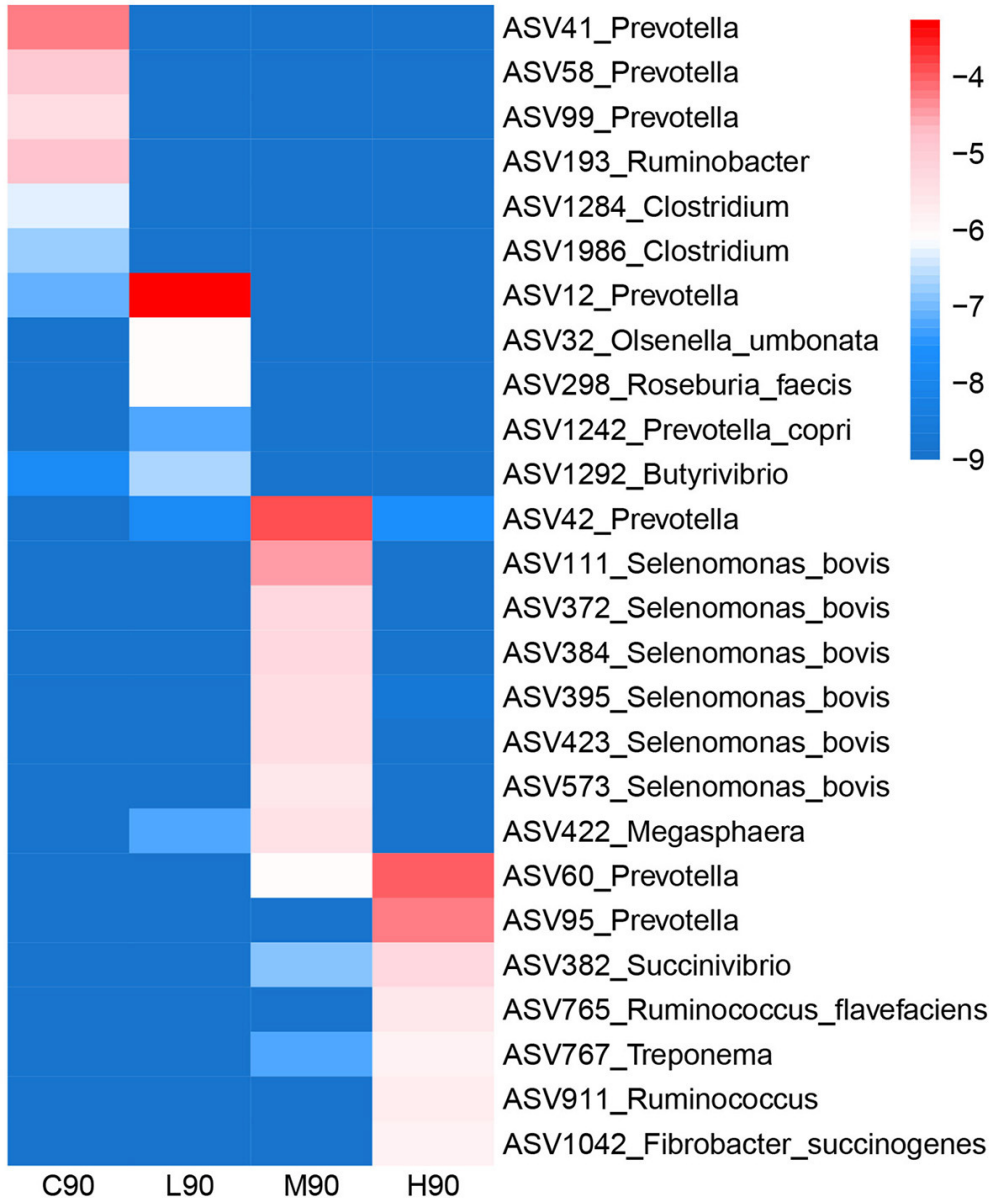
The bacterial ASVs identified by LEfSe analyses were associated with dietary β-hydroxybutyric acid (BHB). A heatmap depicting the signature ASVs for each group identified by the LEfSe algorithm was drawn. The heatmap shows the average relative abundances of ASVs on a log scale. The color of cells from blue to red corresponds to the relative abundance of ASVs from low to high. C90 = controls at 90 days of age; L90: low dose (3 g/d per animal BHB) at 90 days of age; M90: medium dose (6 g/d per animal BHB) at 90 days of age; H90: high dose (9 g/d per animal BHB) at 90 days of age.

The bacterial ASVs distinguishing four different groups were classified using LEfSe analysis, and the relative abundance of these signature ASVs is visualized on a heatmap (Figure 4). *Prevotella* (ASV41, ASV58, and ASV99), *Ruminobacter* (ASV193), and *Clostridium* (ASV1284, ASV1986) were identified as the microbial signature for C90. Bacteria, including *Prevotella* (ASV12), *Prevotella copri* (ASV1242), *Olsenella umbonata* (ASV32), *Roseburia faecis* (ASV293), and *Butyrivibrio* (ASV1292), were enriched in L90. In the M90 group, *Prevotella* (ASV42), *Selenomonas bovis* (ASV111, ASV372, ASV384, ASV395, ASV423, and ASV573), and *Megasphaera* (ASV422) were over-represented. H90 had higher abundances of *Prevotella* (ASV60, ASV95), *Succinivibrio* (ASV382), *Ruminococcus flavefaciens* (ASV765), *Ruminococcus* (ASV911), *Treponema* (ASV767), and *Fibrobacter succinogenes* (ASV1284, ASV1986).

### Network analysis of the rumen microbial interactions

**Figure 5:**
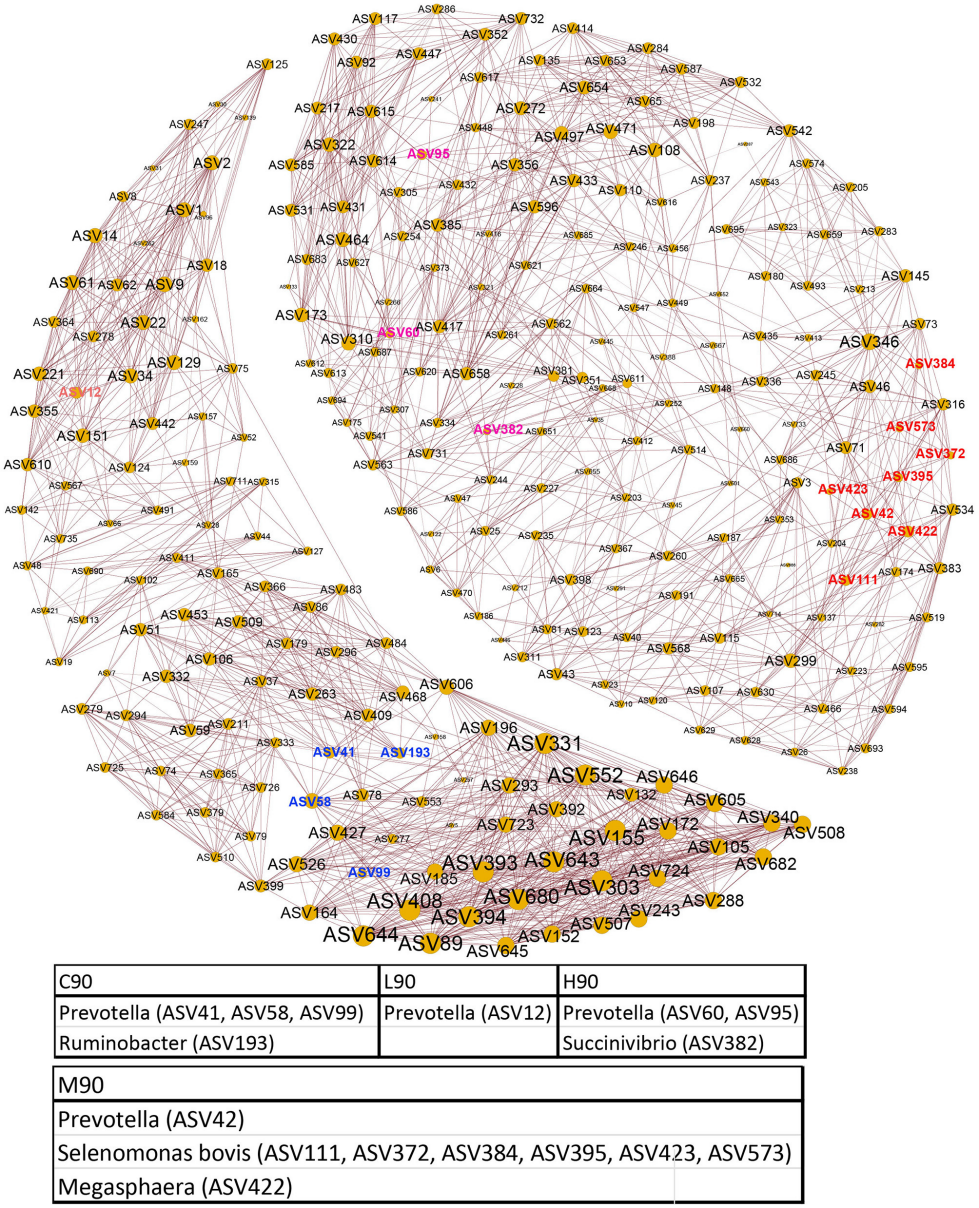
Co-occurrence patterns of rumen bacteria. Co-occurrence networks visualizing significant correlations (ρ > 0.6, *p* < 0.001; indicated with brown lines) between bacterial ASVs in the rumen community.

Rumen microbial interactions were determined using network analysis (Figure 5). We found that the abundance patterns of microbial associations responded to the levels of dietary BHB. Two modules were observed. One of the modules mainly consisted of the microbial signatures for C90 and L90, including *Prevotella* (ASV41, ASV58, and ASV99), *Ruminobacter* (ASV193), and *Prevotella* (ASV12), while another module contained the signature bacterial ASVs for M90 and H90, such as *Prevotella* (ASV42), *Selenomonas bovis* (ASV111, ASV372, ASV384, ASV395, ASV423, and ASV573), *Megasphaera* (ASV422), *Prevotella* (ASV60, ASV95), and *Succinivibrio* (ASV382). In addition, the levels of BHB also drove the changes in microbial interactions of the same module. For example, bacterial ASVs predicting M90 and H90 had more complex correlations within themselves. A similar pattern of bacterial ASV associations between L90 and C90 was also observed.

### Rumen microbiota associated with host phenotypes

To understand how the changed rumen microbiota by BHB links to the host phenotypes, we next performed regression-based random forest using parameters of phenotypes (i.e., body weight, rumen weight, and VFAs) as the outcome and the rumen bacterial ASVs as predictors. The top 50 bacterial ASVs that predict host phenotypes are presented in Supplemenatry Figures S2, S3, and their Spearman correlations were also calculated (Supplemenatry Tables S2, S3). Among the top 50 predictors for body weight and rumen weight, 28 shared bacterial ASVs [i.e., *Prevotella* (ASV22, ASV34, and ASV99) and *Olsenella umbonata* (ASV32, ASV543, and ASV667)] were observed, indicating that improvement in rumen development by microbiota was strongly associated with growth performance of goat kids (Supplementary Figure S2). Moreover, *Prevotella* (ASV99), *Olsenella umbonata* (ASV32), and *Roseburia faecis* (ASV298) identified as signatures by LEfSe were listed. Notably, *Olsenella umbonata* (ASV32) was significantly and positively correlated with body and rumen weight (Supplementary Table S2).

Regarding the bacteria predicting rumen acetate, propionate, and butyrate, four common ASVs were found, including *Prevotella* (ASV34, ASV206, and ASV107) and *Clostridiales* (ASV1483) (Supplemenatry Figure S3). Specifically, more shared bacteria predicted both acetate and propionate, such as *Olsenella umbonata* (ASV32, ASV257), *Prevotella* (ASV22), *Roseburia faecis* (ASV75, ASV298), *Succinivibrio* (ASV14, ASV398), and *Selenomonas bovis* (ASV372). The relative abundances of ASV32, ASV298, ASV14, ASV22, and ASV206 were positively correlated with propionate (Supplementary Table S3). *Succinivibrio* (ASV1) and *Prevotella* (ASV40, ASV61, and ASV115) were classified to predict both propionate and butyrate.

## Discussion

Exogenous ketones and ketone bodies could alter gut microbiota and host physiology (Ang et al., 2020). Most previous studies focus on the beneficial effects of β-hydroxybutyric acid (BHB) on the prevention or improvement of the symptoms of various diseases (Han et al., 2020). However, our recent study in ruminants confirmed that dietary BHB could improve growth performance (Abdelsattar et al., 2022a), which is also validated in this study. This study first determined the rumen microbiota changes that responded to dietary BHB supplementation. We found a significant alteration in the structure and composition of the rumen microbiota in young goats after 2 months of BHB feeding. The levels of exogenous BHB drove the keystone bacterial taxa and their interactions to affect the host phenotypes by influencing biosynthesis of the rumen VFAs in the low-dose BHB group. Our results revealed how BHB affects animal growth via modulation of rumen symbiotic microbiotas and their fermentation, which provide implications for the design of both diet and supplemental interventions in the post-antibiotic era.

BHB, also known as 3-hydroxybutyric acid, is an organic compound and a beta-hydroxy acid with the chemical formula CH3CHCH_2_CO_2_H (Achanta and Rae, 2017). Although BHB and butyrate are chemically similar and have the same functions, BHB is more potent in serving as an energy substrate for ATP generation directly (Cavaleri and Bashar, 2018). To determine the dose of BHB supplementation in young goats, our preliminary experiment (data not shown) was performed to confirm the supplementation level based on a previous human study (Kackley et al., 2020). In this study, we found that BHB improved the rumen development and growth of goat kids, which is in agreement with previous studies on butyrate supplementation (Cavini et al., 2015; Liu et al., 2021). However, we did not observe increases in rumen content and serum BHB concentration. Previous studies in humans and mice found that ketone bodies increased the blood and luminal BHB (Olson et al., 2018; Ang et al., 2020). This might be due to the specific anatomy of rumen epithelium that utilized more BHB or VFAs for its rapid development of the ketogenic capacity in young ruminants (Lane et al., 2002; Abdelsattar et al., 2022b; Wang et al., 2022). In the meantime, a low dose of BHB feeding increased rumen VFAs, including acetate, propionate, and butyrate, while medium and high doses affected them. A previous study confirmed that the addition of D-β-hydroxybutyrate increased butyrogenesis in the human fecal inoculum (Sasaki et al., 2020). Therefore, our results implemented that different concentrations of dietary BHB might affect rumen microbiota and host development in different mechanisms, which should be deeply investigated in future studies.

The low dose of dietary BHB decreased the diversity and richness of the rumen microbiota, although no significant effects of high and medium BHB supplementation were found. Greater concentrations of rumen VFAs and the weight of rumen in the low dose of BHB-feeding goats provided indirect evidence of the diversity changes. However, in our results, significant changes in the rumen microbial membership and structure by the three levels of BHB were observed in goat kids. Alpha diversity measures are the summary statistics of a single population (within-sample diversity), while beta diversity measures are estimates of similarity or dissimilarity between populations (between samples). Thus, the BHB supplementation could influence rumen microbial composition, and with its increasing supplementation dose, its effects on the rumen microbiome community were altered. Considering similar body weight was observed in our experimental groups, it is speculated that low-dose BHB in this study might influence growth performance via rumen microbiotas and its metabolites, while the addition of higher dietary might change the community via increasing or decreasing some bacteria to achieve the same host phenotype. BHB affecting the structure of gut microbiota in humans, sea cucumber *Apostichopus japonicus*, rainbow trout, Siberian sturgeon, and soiny mullet was reported (Najdegerami et al., 2012; Qiao et al., 2019; Sasaki et al., 2020; Liu et al., 2022). Ang et al. (2020) observed that BHB influenced some gut microbiotas in a dose-dependent manner. Moreover, a more significant proportion of excess BHB supplementation may diffuse into the rumen epithelium directly and may be used as energy or a signaling molecule later. Future studies need to deeply characterize the mechanisms of BHB affecting the host development and identify which dose of BHB will have the optimal contribution to a rumen microbial change basis.

Rumen microbiota converts dietary nutrients into VFAs for the maintenance and growth of the host (Zhao et al., 2023). Microbial changes at early life stages have a long-term impact on the rumen ecosystem (Meale et al., 2021). Measurement of BHB impacting early rumen microbiota has beneficial effects on livestock production. An increase of *Prevotella* in the low-dose BHB group was found compared with other groups, which corresponds to the high concentrations of VFAs. Moreover, significant ASV level differences in abundance and dynamics in this genus were identified as signatures for different levels of BHB. For example, *Prevotella* (ASV41, ASV58, and ASV99) was greater in controls, while other ASVs associated with *Prevotella* were enriched in the BHB groups such as ASV12, ASV42, and ASV60, with high abundance in low, medium, and high doses of BHB, respectively. *Prevotella*, a well-known degrader of starch, β glycans, protein, pectin, and hemicellulose, allows its ability to dominate in the rumen under a range of diets since it can use a variety of substrates (Golder et al., 2014). Hence, different ASVs associated with the levels of BHB presented the changes in rumen microbial structure. In addition, some other bacteria were influenced by BHB supplementation. The abundance of *Olsenella* was also increased when low-dose BHB was supplemented with goat kids. *Olsenella* ferments starch and glycogen substrates and produces lactic acid, acetic acid, and formic acid (Mcloughlin et al., 2020), indicating that this bacterium was also affected by BHB. Moreover, we observed that low-dose BHB increased the abundance of *Succinivibrio*. Rumen *Succinivibrio* produces succinate which can be decarboxylated to form acetate, propionate, and butyrate (Hespell, 1992; Iqbal et al., 2018). It reflected that high rumen VFAs in the low-dose BHB group were found in this study. Several ASVs relative to *Selenomonas bovis* were over-represented in the medium-dose BHB group. *Selenomonas* can convert succinate into propionate via the succinate pathway and is sensitive to the ratio of glucogenic to lipogenic nutrients in the diet (Hua et al., 2021). The genus *Treponema* was improved by the medium and high doses of BHB. *Treponema spp*. are a commonly detected bacterial group in the rumen involved in the degradation of soluble fibers and are associated with fatty acid (β-hydroxybutyrate) biosynthesis (Tucci and Martin, 2007; Bekele et al., 2011). Overall, BHB could alter the rumen microbiota, and the dose of BHB could act in different mechanisms to influence the rumen microbiota in young goats.

The fermented products from the gut microbiota directly contribute to the nutrient supply and development of the host (Deng et al., 2023). Modulating the rumen microbiota to improve growth in animals has become a practical approach (Chai et al., 2021). Understanding the correlation between the rumen microbiota and the host phenotype allows us to identify the mechanisms of host–microbe interaction. In our study, we identified the top bacterial taxa that are most positively related to body and rumen weight at 90 days of age. ASV32 associated with *Olsenella umbonata* was positively correlated with both body and rumen weight. Of note, ASV32 was also a signature for the low-dose BHB group and had a positive correlation with rumen propionate concentration. The members of the genus *Olsenella* that ferment carbohydrates predominantly to lactic acid had greater abundance in the gut microbiota of calves consuming high doses of *Macleaya cordata* extract (Stepanchenko, 2021). A study found that the abundance of *Olsenella* in the gut of swine fed a diet supplemented with resistant potato starch was associated with butyrate, caproate, and succinate (Trachsel et al., 2022). Moreover, *Olsenella* was found to be associated with medium-chain fatty acid production (Scarborough et al., 2018). *Olsenella umbonata* was found to have the ability to produce 4-methyl phenol (p-cresol) from p-hydroxyphenylacetate (Li et al., 2015). Another bacterium, *Roseburia faecis* (ASV298), was also positively correlated with propionate, rumen weight, and body weight. Gut *Roseburia spp*. are a part of commensal bacteria, producing short-chain fatty acids, especially butyrate, and affecting colonic motility, immunity maintenance, and anti-inflammatory properties (Tamanai-Shacoori et al., 2017). Several colonic *Roseburia* species in humans could actively metabolize linoleic acid, forming either vaccenic acid or hydroxyl-18:1 fatty acid (Devillard et al., 2007). Therefore, BHB supplementation could affect some core bacteria, and these abundant microbiotas influence ketone body production in the rumen and host development. However, considering no differences in rumen BHB between groups, these ketone bodies might have an alternative way to be utilized by ruminants.

Investigation of the tipping points of dietary BHB dose is critical to obtain the best animal performance and decrease the feeding cost. In this study, low-dose BHB (3 g/day per animal) showed the best rumen fermentation and growth performance, although serum and rumen BHB were unaffected. Moreover, low-dose BHB significantly altered the rumen microbial composition and structure in spite of the greater alteration found in the medium- and high-dose BHB groups. Additionally, our additional study also confirmed positive effects of low dose BHB in rumen papilla development and epithelial gene expression and metabolites, which supplements our findings in this study (Zhuang et al., 2023a). Overall, our results show that partial induction of biosynthesis of rumen VFAs is sufficient to alter the gut microbiota and improve animal development despite the continued intake of BHB, providing support for the importance of ketone bodies in young ruminants. Future studies should deeply investigate the optimal levels of dietary BHB supplementation.

## Conclusion

In conclusion, our study revealed that dietary β-hydroxybutyric acid (BHB) improved growth and rumen environment in goat kids through modulation of the structure and composition of the rumen microbiota. Enhancement of rumen VFA biosynthesis using the low dose of BHB supplementation was confirmed. Different levels of dietary BHB drove the specific rumen bacteria and subsequently influenced the interactions of the keystone taxa, resulting in the alteration of the rumen microbial composition. Taken together, dietary BHB could, in part, alter the rumen microbiota to improve the growth and development of young ruminants. Our study offers a new feed additive to enhance ruminant growth and health in the post-antibiotic era. Furthermore, future studies should determine the optimal dose of BHB supplementation in young ruminants.

## Data availability statement

The data presented in this study are deposited in the NCBI BioProject repository, accession number PRJNA932733.

## Ethics statement

All experimental procedures were approved by the Animal Ethics Committee of the Chinese Academy of Agricultural Sciences (Protocol number: AEC-CAAS-20200605; Approval date: June 3, 2020). The study was conducted in accordance with the local legislation and institutional requirements.

## Author contributions

JC: Conceptualization, Data curation, Formal analysis, Validation, Visualization, Writing—original draft. ZY: Data curation, Investigation, Writing—original draft. JW: Supervision, Writing—review & editing. YK: Data curation, Investigation, Writing—original draft. MA: Investigation, Writing—original draft. WZ: Formal analysis, Writing—original draft. SW: Data curation, Writing—original draft. SY: Investigation, Supervision, Writing—review & editing. FD: Formal analysis, Writing—review & editing. YL: Funding acquisition, Project administration, Resources, Supervision, Writing—review & editing. YZ: Conceptualization, Data curation, Investigation, Supervision, Writing—review & editing. NZ: Funding acquisition, Project administration, Resources, Supervision, Visualization, Writing—review & editing.
