# Supplementary material for: Dietary β-hydroxybutyric acid improves the growth performance of young ruminants based on rumen microbiota and volatile fatty acid biosynthesis
Source: Front Microbiol. 2024 Jan 8;14:1296116. doi: 10.3389/fmicb.2023.1296116 (PMC10801009; doi:10.3389/fmicb.2023.1296116)
Supplement: Supplementary file 1 [file Data_Sheet_1.docx]

Table S1 The chemical composition of basal diets (%, DM basis)

| Item | Milk replacer | Starter diet |
| --- | --- | --- |
| DM | 95.5 | 91.2 |
| ME, MJ/kg | 19.36 | 13.38 |
| OM | 89.0 | 84.2 |
| EE | 16.0 | 3.73 |
| CP | 25.5 | 19.4 |
| Ash | 6.54 | 7.03 |
| NDF | – | 33.2 |
| ADF | – | 15.9 |
| Ca | 1.02 | 0.95 |
| P | 0.66 | 0.70 |

DM = dry matter; ME = metabolizable energy; OM = organic matter; EE = ether extract; CP = crude protein; NDF = neutral detergent fiber; ADF = acid detergent fiber.

ME was calculated by the equations.

Table S2 The Spearman correlation between signature ASVs and growth and rumen weight.

| body weight | coefficient | pvalue |
| --- | --- | --- |
| ASV1236_Succiniclasticum | -0.50 | 0.018 |
| ASV1031_Ruminococcaceae | -0.40 | 0.068 |
| ASV218_Methanobrevibacter | -0.31 | 0.156 |
| ASV1689_Prevotella | -0.48 | 0.024 |
| ASV456_Ruminococcus_albus | -0.43 | 0.043 |
| ASV212_Methanobrevibacter | 0.27 | 0.228 |
| ASV2289_Prevotella | 0.40 | 0.066 |
| ASV122_Succinivibrio | 0.45 | 0.037 |
| ASV260_Prevotella | 0.44 | 0.039 |
| ASV99_Prevotella | -0.39 | 0.076 |
| ASV667_Olsenella_umbonata | -0.43 | 0.048 |
| ASV1181_Prevotella | 0.47 | 0.026 |
| ASV857_Prevotella | 0.55 | 0.009 |
| ASV829_Methanobrevibacter | -0.10 | 0.668 |
| ASV81_Ruminococcus | 0.36 | 0.096 |
| ASV148_Olsenella_umbonata | -0.43 | 0.048 |
| ASV32_Olsenella_umbonata | 0.36 | 0.098 |
| ASV221_Prevotella | 0.37 | 0.093 |
| ASV22_Prevotella | 0.35 | 0.112 |
| ASV34_Prevotella | 0.35 | 0.107 |
| ASV449_Olsenella_umbonata | -0.39 | 0.074 |
| ASV58_Prevotella | -0.11 | 0.635 |
| ASV412_Coriobacteriaceae | 0.15 | 0.498 |
| ASV298_Roseburia_faecis | 0.20 | 0.365 |
| ASV252_Methanobrevibacter | 0.11 | 0.612 |
| ASV67_Acidaminococcus | 0.27 | 0.228 |
| ASV2471_Succiniclasticum | -0.23 | 0.309 |
| ASV1145_Coriobacteriaceae | -0.42 | 0.049 |
| ASV1385_Prevotella | -0.43 | 0.044 |
| ASV2043_Prevotella | 0.50 | 0.018 |
| ASV543_Olsenella_umbonata | -0.26 | 0.235 |
| ASV875_Prevotella | -0.35 | 0.109 |
| ASV1224_S24.7 | 0.19 | 0.403 |
| ASV1527_Lachnospiraceae | 0.35 | 0.109 |
| ASV1804_Prevotella_copri | 0.31 | 0.166 |
| ASV1794_Clostridium_aminophilum | -0.37 | 0.088 |
| ASV1982_Selenomonas_ruminantium | -0.43 | 0.048 |
| ASV1656_Ruminococcus | 0.35 | 0.112 |
| ASV619_Dialister | 0.32 | 0.150 |
| ASV2200_Dialister | 0.33 | 0.130 |
| ASV1109_Ruminococcus_albus | -0.24 | 0.282 |
| ASV446_Megasphaera | 0.15 | 0.503 |
| ASV1293_Butyrivibrio | 0.28 | 0.212 |
| ASV323_Treponema | -0.31 | 0.158 |
| ASV256_Prevotella | -0.24 | 0.278 |
| ASV66_Megasphaera | 0.18 | 0.413 |
| ASV1211_S24.7 | -0.38 | 0.080 |
| ASV1082_Prevotella | -0.41 | 0.057 |
| ASV664_Bacteria | 0.39 | 0.073 |
| ASV2430_Clostridiales | 0.33 | 0.128 |
| rumen weight | | |
| ASV1031_Ruminococcaceae | -0.30 | 0.177 |
| ASV1236_Succiniclasticum | -0.41 | 0.059 |
| ASV449_Olsenella_umbonata | -0.46 | 0.032 |
| ASV218_Methanobrevibacter | -0.21 | 0.344 |
| ASV212_Methanobrevibacter | 0.37 | 0.086 |
| ASV616_Prevotella | -0.39 | 0.074 |
| ASV667_Olsenella_umbonata | -0.48 | 0.025 |
| ASV456_Ruminococcus_albus | -0.43 | 0.048 |
| ASV1689_Prevotella | -0.49 | 0.020 |
| ASV252_Methanobrevibacter | 0.28 | 0.206 |
| ASV122_Succinivibrio | 0.32 | 0.152 |
| ASV1678_Prevotella | 0.39 | 0.070 |
| ASV99_Prevotella | -0.30 | 0.175 |
| ASV543_Olsenella_umbonata | -0.32 | 0.146 |
| ASV22_Prevotella | 0.42 | 0.050 |
| ASV221_Prevotella | 0.46 | 0.032 |
| ASV2200_Dialister | 0.44 | 0.040 |
| ASV323_Treponema | -0.35 | 0.112 |
| ASV2012_Butyrivibrio | 0.44 | 0.039 |
| ASV1527_Lachnospiraceae | 0.44 | 0.039 |
| ASV745_Acidaminococcus | -0.11 | 0.636 |
| ASV32_Olsenella_umbonata | 0.44 | 0.042 |
| ASV34_Prevotella | 0.42 | 0.055 |
| ASV619_Dialister | 0.43 | 0.047 |
| ASV606_Paraprevotellaceae | -0.52 | 0.013 |
| ASV412_Coriobacteriaceae | 0.26 | 0.248 |
| ASV938_Ruminococcus_albus | -0.40 | 0.066 |
| ASV1054_Megasphaera | -0.32 | 0.142 |
| ASV1146_Coriobacteriaceae | -0.33 | 0.128 |
| ASV830_Prevotella_ruminicola | -0.17 | 0.444 |
| ASV195_Succiniclasticum | -0.20 | 0.370 |
| ASV287_Prevotella_ruminicola | -0.26 | 0.238 |
| ASV1293_Butyrivibrio | 0.39 | 0.075 |
| ASV1656_Ruminococcus | 0.45 | 0.037 |
| ASV298_Roseburia_faecis | 0.25 | 0.260 |
| ASV860_Coriobacteriaceae | 0.22 | 0.327 |
| ASV1145_Coriobacteriaceae | -0.39 | 0.070 |
| ASV547_Prevotella | -0.37 | 0.093 |
| ASV1630_Ruminococcus_flavefaciens | -0.37 | 0.090 |
| ASV1333_Ruminococcaceae | -0.31 | 0.158 |
| ASV1734_Prevotella | -0.37 | 0.094 |
| ASV174_S24.7 | 0.16 | 0.473 |
| ASV875_Prevotella | -0.37 | 0.086 |
| ASV1794_Clostridium_aminophilum | -0.26 | 0.235 |
| ASV2262_Ruminococcus_albus | -0.19 | 0.408 |
| ASV1191_Prevotella | -0.31 | 0.166 |
| ASV3_Succinivibrio | -0.17 | 0.451 |
| ASV1079_Bacteroidales | 0.33 | 0.136 |
| ASV2300_Pyramidobacter | -0.40 | 0.066 |
| ASV1385_Prevotella | -0.34 | 0.116 |

Table S3 The Spearman correlation between signature ASVs and rumen VFAs.

| Acetic | coefficient | pvalue |
| --- | --- | --- |
| ASV367_Prevotella | 0.53 | 0.011 |
| ASV2047_Treponema | -0.33 | 0.129 |
| ASV257_Olsenella_umbonata | 0.33 | 0.132 |
| ASV75_Roseburia_faecis | 0.09 | 0.695 |
| ASV601_Succiniclasticum | 0.06 | 0.783 |
| ASV1483_Clostridiales | -0.46 | 0.033 |
| ASV372_Selenomonas_bovis | -0.20 | 0.383 |
| ASV206_Prevotella | 0.27 | 0.216 |
| ASV395_Selenomonas_bovis | -0.41 | 0.057 |
| ASV573_Selenomonas_bovis | -0.41 | 0.057 |
| ASV1733_Ruminococcus_flavefaciens | 0.21 | 0.353 |
| ASV547_Prevotella | 0.20 | 0.379 |
| ASV2290_Prevotella | 0.30 | 0.175 |
| ASV398_Succinivibrio | 0.37 | 0.086 |
| ASV812_Roseburia_faecis | -0.28 | 0.212 |
| ASV2262_Ruminococcus_albus | 0.60 | 0.003 |
| ASV652_Prevotella | 0.58 | 0.004 |
| ASV46_S24.7 | -0.36 | 0.096 |
| ASV435_Olsenella_umbonata | -0.42 | 0.051 |
| ASV22_Prevotella | 0.24 | 0.279 |
| ASV543_Olsenella_umbonata | -0.39 | 0.076 |
| ASV930_Ruminococcaceae | -0.43 | 0.046 |
| ASV830_Prevotella_ruminicola | -0.28 | 0.204 |
| ASV413_Treponema | -0.39 | 0.076 |
| ASV2491_Coriobacteriaceae | -0.41 | 0.056 |
| ASV316_S24.7 | -0.36 | 0.100 |
| ASV298_Roseburia_faecis | 0.27 | 0.227 |
| ASV994_Prevotella | 0.57 | 0.005 |
| ASV1146_Coriobacteriaceae | -0.44 | 0.042 |
| ASV73_S24.7 | -0.39 | 0.070 |
| ASV178_Megasphaera | -0.23 | 0.297 |
| ASV34_Prevotella | 0.16 | 0.483 |
| ASV32_Olsenella_umbonata | 0.13 | 0.554 |
| ASV1473_Prevotella | -0.28 | 0.213 |
| ASV1007_Prevotella | 0.55 | 0.008 |
| ASV45_Succinivibrio | 0.50 | 0.018 |
| ASV129_Prevotella | 0.07 | 0.743 |
| ASV1181_Prevotella | 0.24 | 0.290 |
| ASV1223_Succiniclasticum | 0.08 | 0.720 |
| ASV1666_Prevotella | 0.15 | 0.500 |
| ASV108_S24.7 | 0.35 | 0.108 |
| ASV14_Succinivibrio | 0.27 | 0.233 |
| ASV237_Prevotella | 0.60 | 0.003 |
| ASV762_Prevotella | -0.44 | 0.041 |
| ASV145_Prevotella | -0.39 | 0.071 |
| ASV221_Prevotella | 0.14 | 0.545 |
| ASV289_Acidaminococcus | 0.16 | 0.469 |
| ASV2471_Succiniclasticum | -0.40 | 0.067 |
| ASV71_Prevotella | -0.40 | 0.065 |
| ASV606_Paraprevotellaceae | -0.40 | 0.062 |
| Propionate | | |
| ASV398_Succinivibrio | 0.40 | 0.067 |
| ASV22_Prevotella | 0.54 | 0.009 |
| ASV367_Prevotella | 0.48 | 0.022 |
| ASV34_Prevotella | 0.35 | 0.105 |
| ASV32_Olsenella_umbonata | 0.37 | 0.092 |
| ASV12_Prevotella | 0.38 | 0.085 |
| ASV43_Ruminococcus | 0.43 | 0.047 |
| ASV221_Prevotella | 0.44 | 0.043 |
| ASV298_Roseburia_faecis | 0.56 | 0.007 |
| ASV206_Prevotella | 0.53 | 0.012 |
| ASV115_Prevotella | 0.17 | 0.452 |
| ASV1236_Succiniclasticum | -0.29 | 0.187 |
| ASV75_Roseburia_faecis | 0.13 | 0.575 |
| ASV1405_Ruminococcus_flavefaciens | -0.31 | 0.156 |
| ASV2270_Succiniclasticum | -0.35 | 0.111 |
| ASV14_Succinivibrio | 0.54 | 0.009 |
| ASV2436_Prevotella_copri | 0.54 | 0.009 |
| ASV61_Prevotella | 0.59 | 0.004 |
| ASV994_Prevotella | 0.54 | 0.009 |
| ASV1_Succinivibrio | 0.50 | 0.017 |
| ASV67_Acidaminococcus | 0.34 | 0.121 |
| ASV1031_Ruminococcaceae | -0.21 | 0.345 |
| ASV1483_Clostridiales | -0.37 | 0.093 |
| ASV527_Clostridiales | 0.03 | 0.897 |
| ASV1007_Prevotella | 0.51 | 0.015 |
| ASV9_Succinivibrio | 0.19 | 0.395 |
| ASV218_Methanobrevibacter | -0.15 | 0.512 |
| ASV40_Prevotella | 0.26 | 0.236 |
| ASV606_Paraprevotellaceae | -0.38 | 0.077 |
| ASV2407_Coriobacteriaceae | 0.46 | 0.032 |
| ASV1224_S24.7 | 0.58 | 0.005 |
| ASV2185_Ruminococcus_albus | -0.29 | 0.188 |
| ASV257_Olsenella_umbonata | 0.30 | 0.172 |
| ASV849_Dialister | 0.54 | 0.009 |
| ASV1630_Ruminococcus_flavefaciens | -0.27 | 0.228 |
| ASV2295_Selenomonas_bovis | 0.50 | 0.018 |
| ASV1186_Prevotella | -0.29 | 0.185 |
| ASV767_Treponema | -0.29 | 0.193 |
| ASV2457_Succinivibrio | 0.55 | 0.008 |
| ASV1491_Acidaminococcus | 0.48 | 0.025 |
| ASV372_Selenomonas_bovis | -0.07 | 0.763 |
| ASV2012_Butyrivibrio | 0.38 | 0.082 |
| ASV2471_Succiniclasticum | -0.29 | 0.192 |
| ASV259_Acidaminococcus | 0.35 | 0.110 |
| ASV1914_Clostridiales | 0.54 | 0.009 |
| ASV1289_Acidaminococcus | 0.22 | 0.336 |
| ASV2501_S24.7 | 0.18 | 0.422 |
| ASV123_Succinivibrio | 0.45 | 0.035 |
| ASV1796_Bacteroidales | -0.25 | 0.261 |
| ASV1707_Roseburia_faecis | 0.27 | 0.225 |
| Butyrate | | |
| ASV206_Prevotella | 0.34 | 0.123 |
| ASV40_Prevotella | 0.37 | 0.090 |
| ASV2067_Prevotella | 0.66 | 0.001 |
| ASV115_Prevotella | 0.31 | 0.164 |
| ASV187_Prevotella | 0.36 | 0.095 |
| ASV20_Olsenella_umbonata | 0.16 | 0.478 |
| ASV652_Prevotella | 0.64 | 0.001 |
| ASV41_Prevotella | -0.23 | 0.310 |
| ASV1483_Clostridiales | -0.39 | 0.077 |
| ASV193_Ruminobacter | -0.32 | 0.142 |
| ASV2262_Ruminococcus_albus | 0.62 | 0.002 |
| ASV2052_Ruminococcus_albus | -0.04 | 0.843 |
| ASV61_Prevotella | 0.23 | 0.309 |
| ASV1236_Succiniclasticum | 0.13 | 0.562 |
| ASV165_Prevotella | -0.25 | 0.267 |
| ASV707_Clostridiales | 0.18 | 0.418 |
| ASV263_Prevotella | -0.33 | 0.140 |
| ASV421_Clostridiales | 0.11 | 0.635 |
| ASV796_Prevotella | -0.09 | 0.688 |
| ASV241_Bacteroidales | -0.13 | 0.557 |
| ASV1044_Prevotella | 0.23 | 0.314 |
| ASV468_Prevotella_ruminicola | -0.36 | 0.100 |
| ASV1283_Methanobrevibacter | 0.06 | 0.785 |
| ASV506_Prevotella | 0.37 | 0.091 |
| ASV1305_Veillonellaceae | 0.32 | 0.141 |
| ASV509_Ruminobacter | -0.35 | 0.107 |
| ASV133_Clostridiaceae | -0.30 | 0.173 |
| ASV1638_Selenomonas_bovis | -0.31 | 0.163 |
| ASV251_Prevotella | 0.26 | 0.247 |
| ASV1032_Prevotella | 0.05 | 0.835 |
| ASV2230_Methanobrevibacter | 0.50 | 0.017 |
| ASV1282_TG5 | 0.23 | 0.297 |
| ASV453_Ruminobacter | -0.25 | 0.270 |
| ASV1_Succinivibrio | 0.21 | 0.337 |
| ASV2505_Prevotella | 0.38 | 0.085 |
| ASV665_Clostridiales | -0.29 | 0.193 |
| ASV1812_Bacteroidales | -0.29 | 0.193 |
| ASV106_Ruminobacter | -0.21 | 0.344 |
| ASV1004_Methanobrevibacter | 0.45 | 0.035 |
| ASV151_Prevotella | 0.02 | 0.924 |
| ASV237_Prevotella | 0.67 | 0.001 |
| ASV262_Prevotella | -0.01 | 0.959 |
| ASV395_Selenomonas_bovis | -0.36 | 0.099 |
| ASV1007_Prevotella | 0.35 | 0.105 |
| ASV331_Selenomonas_ruminantium | 0.27 | 0.230 |
| ASV34_Prevotella | 0.03 | 0.888 |
| ASV1428_Prevotella | 0.38 | 0.082 |
| ASV1796_Bacteroidales | 0.13 | 0.563 |
| ASV1978_Sharpea | 0.26 | 0.235 |
| ASV114_Acidaminococcus | 0.03 | 0.893 |


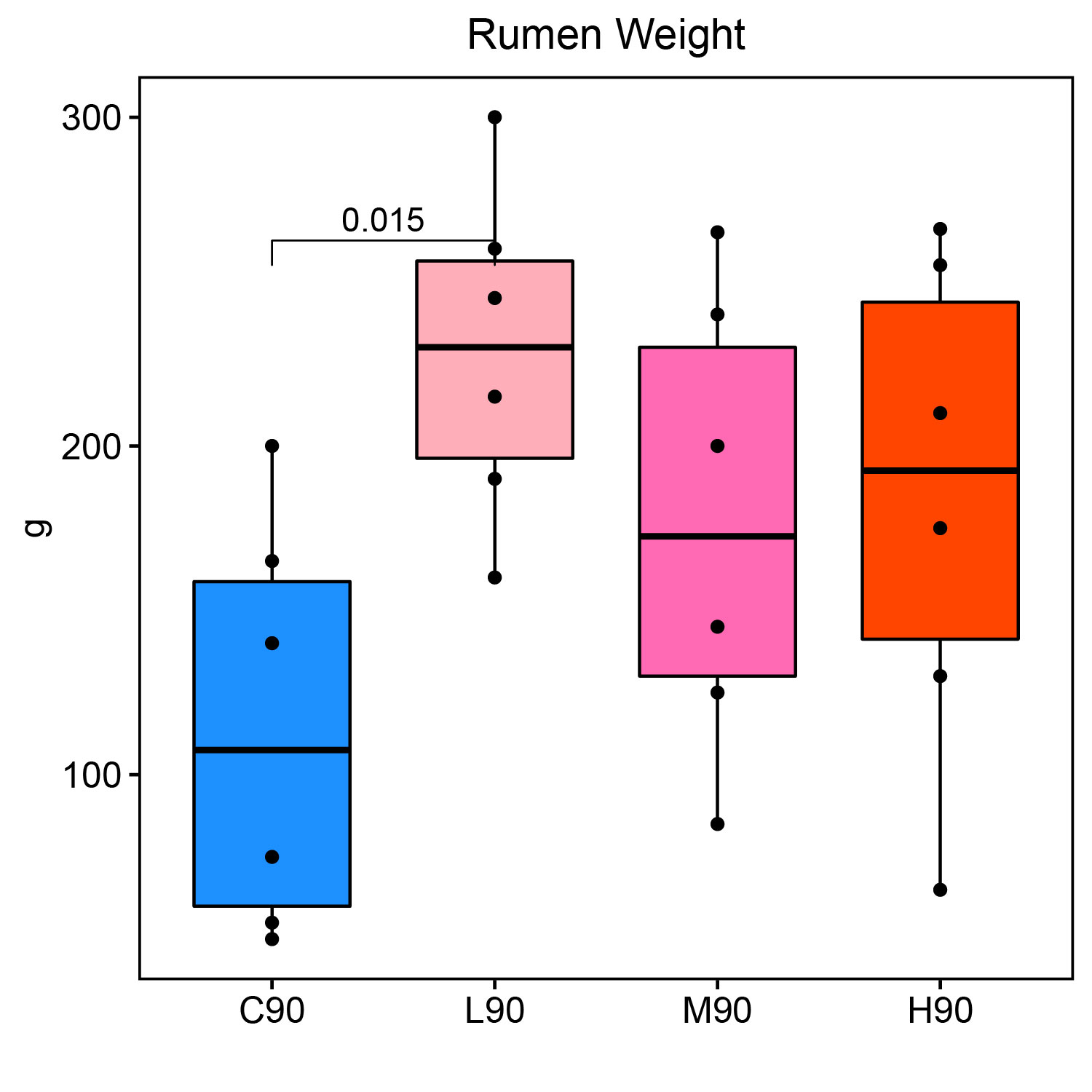


Figure S1 Dietary β-hydroxybutyric acid (BHB) improved rumen weight in goat kids.

C90 = controls at 90 days of age; L90: low dose (3 g/d per animal BHB) at 90 days of age; M90: medium dose (6 g/d per animal BHB) at 90 days of age; H90: high dose (9 g/d per animal BHB) at 90 days of age.


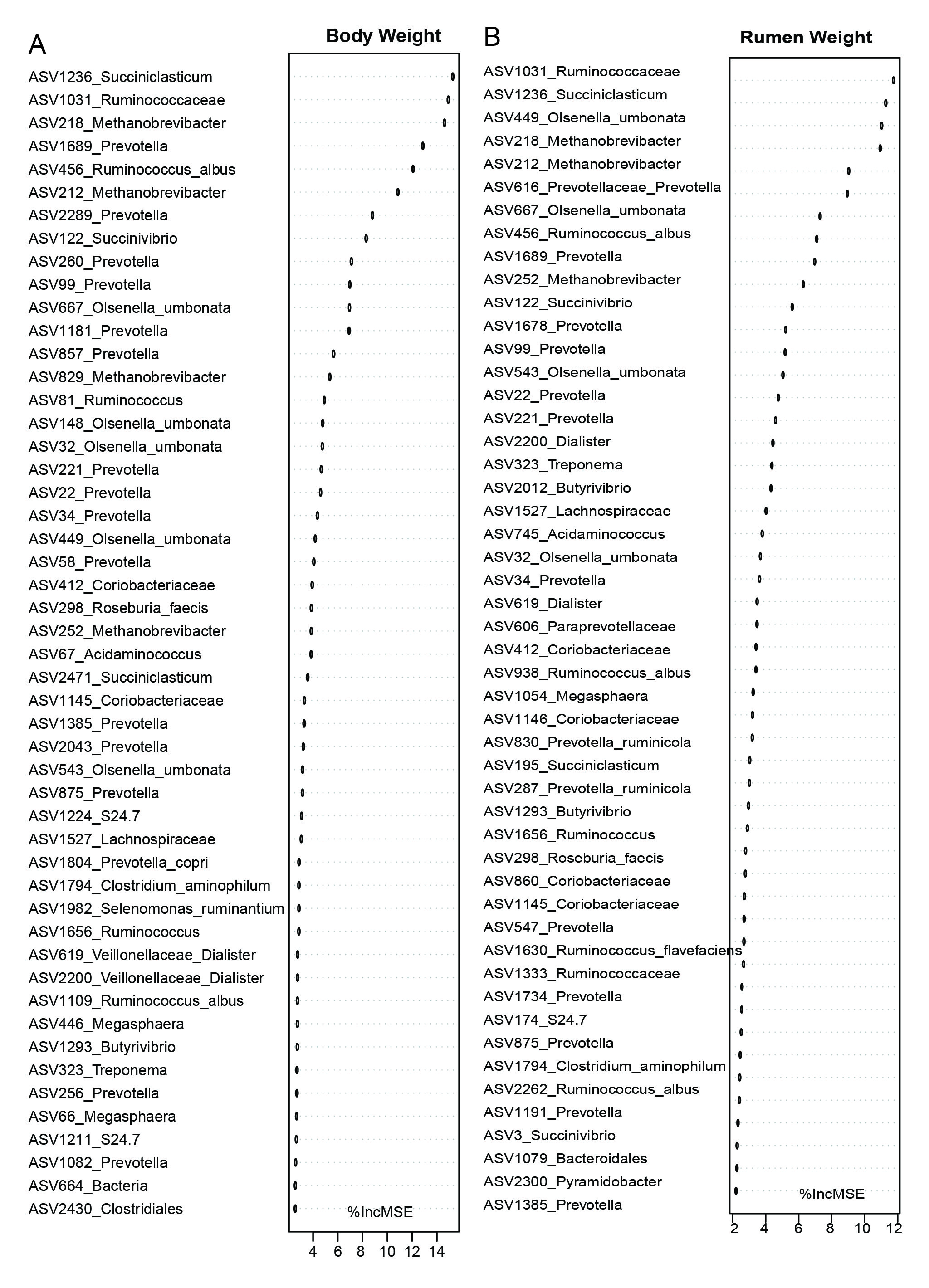


Figure S2 Growth and rumen weight related ASVs. The top 50 body and rumen weight related bacteria in the rumen of goat kids were selected using regression-based random forest algorithm in R.


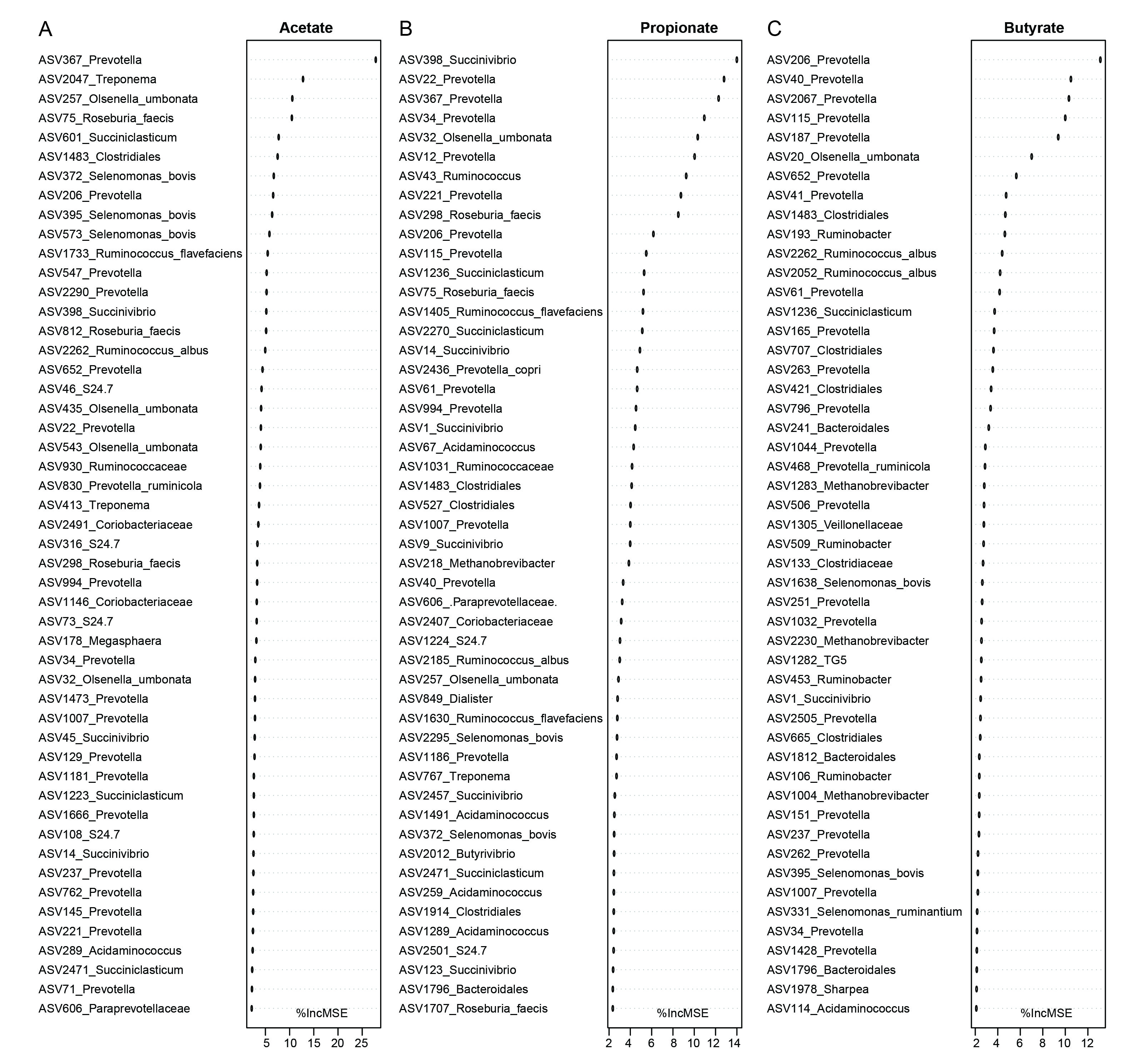


Figure S3 Rumen VFAs related ASVs. The top 50 VFAs (e.g., acetate, propionate, and butyrate) related bacteria in the rumen of goat kids were selected using regression-based random forest algorithm in R.
